# Etymologia: Babesia [bə-be′ ze-ə]

**DOI:** 10.3201/eid1505.999999

**Published:** 2009-05

A genus of protozoa of the order Piroplasmida, named for Victor Babès (1854–1926), a Romanian bacteriologist who discovered a parasitic sporozoon in ticks in 1885. The parasites occur within the erythrocytes of various vertebrates and cause babesiosis, a tick-borne infection of domestic animals and humans.

Babès was also a coauthor of the first text on bacteriology (Bacteria and Their Role in the Anatomy and Pathological Histology of Contagious Diseases, with French scientist A.V. Cornil); the first to demonstrate the presence of tuberculous bacilli in the urine of infected patients; a founder of serum therapy; and the first to introduce rabies vaccination to Romania.

